# Care Me Too: A Mobile App for Engaging Chinese Immigrant Caregivers in Self-Care and Its User-Experience Testing

**DOI:** 10.1093/geroni/igaa057.329

**Published:** 2020-12-16

**Authors:** Mandong Liu, Tongge Jiang, Kexin Yu, Shinyi Wu, Maryalice Jordan-Marsh, Iris Chi

**Affiliations:** University of Southern California, Los Angeles, California, United States

## Abstract

Caregiving and self-care are challenging for Chinese immigrants in the U.S. due to limited accessible support and resources. The team developed a Care Me Too app for engaging Chinese immigrant caregivers in self-care and conducted a user-experience test to assess its usability and acceptability. The aims of this paper are to report the testing results and develop guidelines to support mHealth app design for immigrant caregiver populations. Twenty-two Chinese caregivers participated in the test, which consisted of two parts: in-lab testing and one-week at-home testing. In-depth face-to-face interviews and follow-up phone interviews were used to test the app’s usability and acceptability and solicit participants’ feedback for app design and functions. Directed content analysis was used to analyze testing transcripts. Participants reported uniformly positive ratings of usability and acceptability of the app and provided detailed suggestions for app improvement. We generated some mHealth app design guidelines, including weighing flexibility vs. targeting majority preferences, increasing text sizes, using colors effectively, providing engaging and playful visual designs and functions, simplifying navigation, simplifying login process, design functions to cater to the population’s context, etc. We concluded that culturally and linguistically appropriate mHealth apps are appealing to immigrant caregivers for health promotion.

